# Baseline Quantitative Hepatitis B Core Antibody Titer Is a Predictor for Hepatitis B Virus Infection Recurrence After Orthotopic Liver Transplantation

**DOI:** 10.3389/fimmu.2021.710528

**Published:** 2021-10-27

**Authors:** Bin Lou, Guanghua Ma, Feifei LV, Quan Yuan, Fanjie Xu, Yuejiao Dong, Sha Lin, Yajun Tan, Jie Zhang, Yu Chen

**Affiliations:** ^1^ Department of Laboratory Medicine, The First Affiliated Hospital, College of Medicine, Zhejiang University, Hangzhou, China; ^2^ Key Laboratory of Clinical In Vitro Diagnostic Techniques of Zhejiang Province , Hangzhou, China; ^3^ Institute of Laboratory Medicine, Zhejiang University, Hangzhou, China; ^4^ National Institute of Diagnostics and Vaccine Development in Infectious Disease, School of Life Science, Xiamen University, Xiamen, China

**Keywords:** qHBcAb, liver transplantation, HBV recurrence, sustained HBV loss, qHBsAg

## Abstract

**Objective:**

Hepatitis B virus (HBV) reinfection is a serious complication that arise in patients who undergo hepatitis B virus related liver transplantation. We aimed to use biomarkers to evaluate the HBV reinfection in patients after orthotopic liver transplantation.

**Methods:**

Seventy-nine patients who underwent liver transplantation between 2009 and 2015 were enrolled, and levels of biomarkers were analyzed at different time points. Cox regression and receiver operating characteristic (ROC) curves of different markers at baseline were used to analyze sustained hepatitis B surface antigen (HBsAg) loss. The Kaplan-Meier method was used to compare the levels of the biomarkers.

**Results:**

Among the 79 patients, 42 sustained HBsAg loss with a median time of 65.2 months (12.0-114.5, IQR 19.5) after liver transplantation and 37 patients exhibited HBsAg recurrence with a median time of 8.8 (0.47-59.53, IQR 19.47) months. In the ROC curve analysis, at baseline, 4.25 log_10_ IU/mL qHBcAb and 2.82 log_10_ IU/mL qHBsAg showed the maximum Youden’s index values with area under the curves (AUCs) of 0.685and 0.651, respectively. The Kaplan-Meier method indicated that qHBsAg and quantitative antibody against hepatitis B core antigen (qHBcAb) levels in the two groups were significantly different (*p* = 0.031 and 0.006, respectively). Furthermore, the Cox regression model confirmed the predictive ability of qHBcAb at baseline (AUC = 0.685).

**Conclusion:**

Lower pretransplantation qHBcAb is associated with HBV infection. The baseline concentration of qHBcAb is a promising predictor for the recurrence of HBV in patients undergoing liver transplantation and can be used to guide antiviral treatment for HBV infection.

## Introduction

Hepatitis B virus (HBV) infection is a global health problem. Chronic hepatitis B (CHB) is a major disease that threatens human health, especially in southeast Asia and Africa. According to clinical statistics, the global prevalence of hepatitis B surface antigen (HBsAg) was 3.9% in 2016, corresponding to 291 million infections (1). Additionally, nearly 25% of chronic HBV carriers develop terminal stage liver-related diseases including chronic hepatitis, cirrhosis, and primary hepatocellular carcinoma, causing 0.8 million deaths annually (2, 3). Liver transplantation (LT) is an established treatment option for serious liver disease caused by HBV infection. The data from the European Liver Transplant Registry database (ELTR) verified that viral hepatitis B was the most common indication (9.8%) of LT from 2007 to 2017 (4). HBV recurrence is a risk factor for patients who have undergone LT and leads to grievous failure of treatment and poor prognosis (5, 6). The HBV reinfection rate was higher than 90% (7) and the 2-year survival rate was only 50% (8) before the application of immune globulin and antiviral drugs. In recent years, the therapeutic strategy of nucleos(t)ide analogs (NAs) combined with immune globulin has proven useful in preventing HBV reinfection after LT (9, 10). However, this strategy does not completely protect against future recurrence of HBV infection. HBV reinfection is not only dependent on residual viral infection in extrahepatic organs, but also individual immune responses. Therefore, it is necessary to find satisfactory biomarkers that reflect specific immune responses to HBV to predict the recurrence of HBsAg.

Antibody against hepatitis B core antigen (HBcAb) is a widespread biomarker in patients with HBV infection and can be present in current or previous infections. Routine serum screening for HBcAb and other HBV-related biomarkers has been performed in some countries with a high HBV infection rate. Nevertheless, for some patients, HBcAb may be the only serological marker of HBV infection in serum (11, 12), and “HBcAb only” status may reflect occult HBV infection (13). Additionally, HBcAb alone can be used to evaluate the risk for HBV reactivation in patients who have received antiviral treatment that may lead to immunosuppression, patients with human immunodeficiency virus (HIV) infection or chronic hepatitis C virus (HCV) infection (14, 15).

One study revealed that HBcAb plays a valuable role in CHB disease by inhibiting or clearing HBV (16). Thus, patients with a high titer of HBcAb before therapy had a stronger acquired immune response that was related to a satisfactory outcome after antiviral treatment (17).

Owing to the inferior performance of the competitive immunoassay, current commercially available HBcAb assays have a narrow linearity range. A novel immunoassay for qualitative HBcAb based on the double-antigen sandwich enzyme-linked immunosorbent assay method has demonstrated sensitivity and specificity (18).

In this retrospective longitudinal study, we aimed to analyze the clinical value of qHBcAb, qHBsAg and other viral marker concentrations, focusing on the outcome of patients who underwent LT.

## Materials and Methods

### Patients

Overall, 363 patients who underwent HBV-related LT between 2009 and 2015 at the First Affiliated Hospital, College of Medicine, Zhejiang University were enrolled in this study. The patients selected were also negative for HCV infection, HIV infection, and other chronic diseases. Serum samples were collected and stored at -80°C until use. This study was reviewed and approved by the Ethics Committees of the First Affiliated Hospital, College of Medicine, Zhejiang University. This study followed the 1964 Helsinki Declaration and its later amendments. Prior informed consent was obtained from the enrolled patients or their legal guardians.

### Post-Transplant Therapy

Antiviral prophylaxis was administered to all recipients, and they were treated with NAs in combination with low-dose hepatitis B immunoglobulin (HBIG). Briefly, 2000 IU HBIG was intravenously injected during the anhepatic phase, followed by 800 IU daily intramuscular administration for the first week and then weekly for 3 weeks, and monthly thereafter.

All patients received a Tacrolimus based steroid-sparing or steroid-free immunosuppression regimen. Tacrolimus and mycophenolate mofetil (750 mg every 12 h) were administered from the first post-operative day. The target blood trough level of tacrolimus was 7-10 ng/mL for the first postoperative month and was aimed at 5-7 ng/mL thereafter.

### Measurement of qHBcAb Concentration in Serum

The qHBcAb level in serum was measured using a newly developed double-sandwich immunoassay (100–100 000 IU/mL; Wantai, China), calibrated using the World Health Organization standard (NIBSC, UK) (19, 20). QHBcAb was measured at baseline, with the sought time of HBsAg‐positive status before LT being at least two weeks, and then at different time points after LT.

### Measurement of qHBsAg, qHBeAg, HBcAb and HBV DNA Concentrations in the Serum

Serum levels of qHBsAg, qHBeAg and HBcAb were measured using a chemiluminescence microparticle immunoassay (CMIA) on an Abbott Architect I4000 automated analyzer (Abbott Laboratories, Chicago, IL, USA). HBV DNA levels in serum were measured using Qiagen PCR kits (Hilden, Germany) on ABI 7500 qRT-PCR System (ABI Laboratories, USA) according to the manufacturer’s instructions. The linear detection range was 3 - 7 log_10_ IU/mL, with a correlation coefficient of the standard curve >0.995. Biomarkers were measured before LT and at different time points after LT.

### Statistical Analysis

Chi−squared and Mann−Whitney U tests were performed as appropriate. The accuracy of serum qHBcAb and qHBsAg levels predicting HBsAg recurrence was determined by applying the receiver operating characteristic (ROC) curve analysis. HBV reinfection was assessed using Kaplan-Meier survival analysis. The risk of HBV reinfection after LT was determined using the Cox regression model. Statistical significance was set at *p* < 0.05. Statistical analysis and presentation were performed using SPSS software (version 23.0; SPSS, Chicago, IL, USA). Graphical analysis and data presentation were performed using GraphPad Prism version 5.0 and R version 4.0 (R Foundation).

## Results

### Characteristics of Patients

A total of 363 patients were enrolled in this study, 79 patients were selected retrospectively, and 284 patients were excluded including 44 patients with HBsAg-negative status, 53 patients with underlying diseases or other infectious diseases, 18 patients receiving an HBV-positive graft, 17 patients who died post LT, 5 patients who underwent retransplantation and 147 patients without sufficient serum samples or integrated follow-up time points (**Supplementary Figure 1**). Among the 79 enrolled patients, 37 exhibited HBsAg recurrence with a median time of 8.8 (0.47 - 59.53, IQR 19.47) months, and 42 sustained HBsAg loss with a median follow-up time of 65.2 months (12.0 - 114.5, IQR 19.5) after LT.

The clinical characteristics of patients at baseline are shown in **Table 1** for patients who retained sustained HBsAg loss or achieved HBsAg recurrence during the follow-up period. There were no significant differences in sex, age and treatment received between the two groups (*p* = 0.672, 0.089 and 0.515, respectively). The characteristics of qHBcAb and qHBsAg were significantly different between the groups (*p* = 0.037 and 0.023, respectively). Compared with HBcAb measured by Abbott Architect CMIA (indirect method), the difference in qHBcAb measured by double-sandwich immunoassay was more significant between the observed groups (*p* = 0.023 vs 0.743). The titers of HBV DNA, qHBeAg, alanine aminotransferase (ALT) and aspartate aminotransferase (AST) were not significantly different among the compared groups (*p* = 0.394, 0.732, 0.453 and 0.146, respectively).

**Table 1:** Characteristics of enrolled patients at baseline.

Characteristics	HBsAg recurrence	Sustained HBsAg loss	*p*
	in follow-up time	after LT	
	(n = 37)	(n = 42)	
Male, n (%)	33 (89%)	37 (88%)	*0.672^a^*
Age (years) at baseline before	54 (28 - 67)	48 (29 - 66)	*0.089^b^*
liver transformation, median (range)			
Treatment received			*0.515^a^*
LAM/HBIG	17 (45.95%)	22 (52.38%)	
ADV/LAM/HBIG	10 (27.03%)	8 (19.05%)	
ETV/HBIG	6 (16.22%)	4 (9.52%)	
ETV/LAM/HBIG	4 (10.81%)	8 (19.05%)	
HBV related disease before LT			*0.293^a^*
HCC	22 (59.46%)	20 (47.62%)	
Non-HCC	15 (40.54%)	22 (52.38%)	
qHBsAg (log_10_ IU/mL), median (range)	2.80 (-0.31 - 3.95)	2.34 (-0.89 - 4.22)	0.037^c^*
qHBeAg (log_10_ 0.18 PEIU/mL), median (range)	-0.36 (0-1.57)	-0.39 (0-1.46)	*0.732^c^*
HBcAb (S/CO)	10.38 (0.22 - 47.27)	10.73 (1.88 - 14.16)	*0.743^c^*
qHBcAb (log_10_ IU/mL), median (range)	3.51 (1.06 - 4.85)	4.01 (2.12 - 5.12)	0.023^c^*
HBV DNA (positive%)	12 (32.43%)	10 (23.81%)	*0.394^a^*
ALT (IU/mL), median (range)	39 (14 - 1149)	43 (17 - 221)	*0.453^c^*
AST (IU/mL), median (range)	59 (22 - 1535)	53 (21 - 355)	*0.146^c^*

LAM, lamivudine; ADV, adefovir; ETV, entecavir; HBIG, hepatitis B immunoglobulin; ALT, alanine aminotransferase; AST, aspartate aminotransferase; HCC, hepatocellular carcinoma.

^a^Chi-Square test. ^b^Independent samples t test. ^c^Mann-Whitney U test. *p < 0.05.

### Kinetics of qHBcAb and qHBsAg in HBsAg Recurrence and Sustained HBsAg Loss Groups

**Figure 1:**
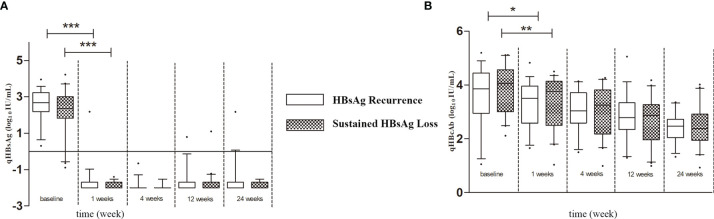
**(A)** The Kinetics of qHBsAg in HBsAg recurrence and sustained HBsAg loss groups. **(B)** The Kinetics of qHBcAb in HBsAg recurrence and sustained HBsAg loss groups. Box plots showing the median, interquartile range and absolute range of qHBcAb and qHBsAg at baseline and at 1 week, 4 weeks, 12 weeks and 24 weeks in groups (blank box plots: HBsAg recurrence group; latticed box: sustained HBsAg loss group. *p<0.05; **p<0.01; ***p<0.001).

As shown in **Figure 1**, during baseline to 1 week after LT, both qHBsAg and qHBcAb were significantly decreased in both sustained HBsAg loss group and HBsAg recurrence group (*p* < 0.001 and *p* < 0.05). However, the changes in qHBcAb and qHBsAg in the two groups were not significantly different after 1 week. At each time point between baseline and 24 weeks after LT, the titer of qHBcAb in the sustained HBsAg loss group was higher than that in the HBsAg recurrence group.

### Comparison of Potential Risk Factors at Baseline Using the Cox Regression Model

To further evaluate the potential risk factors associated with HBV recurrence after LT, Cox regression analysis was performed for age, sex, ALT, HBV DNA, qHBsAg, qHBeAg and qHBcAb. As shown in **Table 2** and **Supplementary Figure 2**, the qHBcAb level at baseline was a strong predictor for HBsAg recurrence after LT, with both the univariate and multivariate analyses (crude HR: 0.40; 95% CI: 0.20-0.78; *p* = 0.01 vs adjusted HR 0.42; 95% CI: 0.19-0.94; *p* = 0.04).

**Table 2:** Risk factors for HBV recurrence after liver transplantation.

Variable	Univariate analysis				Multivariate analysis			
	B	SE	*p*	Crude HR	B	SE	*p*	Adjusted HR
qHBsAg^a,d^	0.70	0.33	0.03^*^	2.01 (1.05-3.86)	0.59	0.37	0.11	1.80 (0.87-3.72)
qHBcAb^b,d^	-0.92	0.35	0.01^*^	0.40 (0.20-0.78)	-0.87	0.41	0.04^*^	0.42 (0.19-0.94)
Age^e^	0.03	0.02	0.07	1.03 (1.00-1.06)	0.02	0.02	0.35	1.02 (0.98-1.05)
Sex^c^	0.13	0.42	0.76	1.13 (0.50-2.59)	-0.37	0.47	0.44	0.69 (0.28-1.74)
ALT^e^	0.00	0.00	0.18	1.00 (1.00-1.01)	0.00	0.00	0.83	0.99 (0.98-1.00)
AST^e^	0.00	0.00	0.09	1.00 (1.00-1.01)	0.00	0.00	0.17	1.00 (0.99-1.02)
HBV DNA^c,d^	0.26	0.35	0.47	1.29 (0.65-2.57)	0.59	0.43	0.17	1.79 (0.77-4.23)
qHBeAg^e^	0.02	0.02	0.38	1.02 (0.97-1.07)	0.01	0.02	0.77	1.01 (0.96-1.06)

The variables included in the Cox regression analysis were age, sex (female vs male), and baseline ALT, HBV DNA, qHBsAg, qHBeAg and qHBcAb levels.

^a^qHBsAg ≤ 2.82 vs > 2.82 log10 IU/mL, ^b^HBcAb < 4.25 vs ≥ 4.25 log10 IU/mL, ^c^HBV DNA ≤ 3.0 vs > 3.0 log10 IU/mL, ^d^Categorical variable, ^e^Continuous variable, *p < 0.05.

### QHBcAb and qHBsAg as Predictors of HBsAg Recurrence

**Figure 2:**
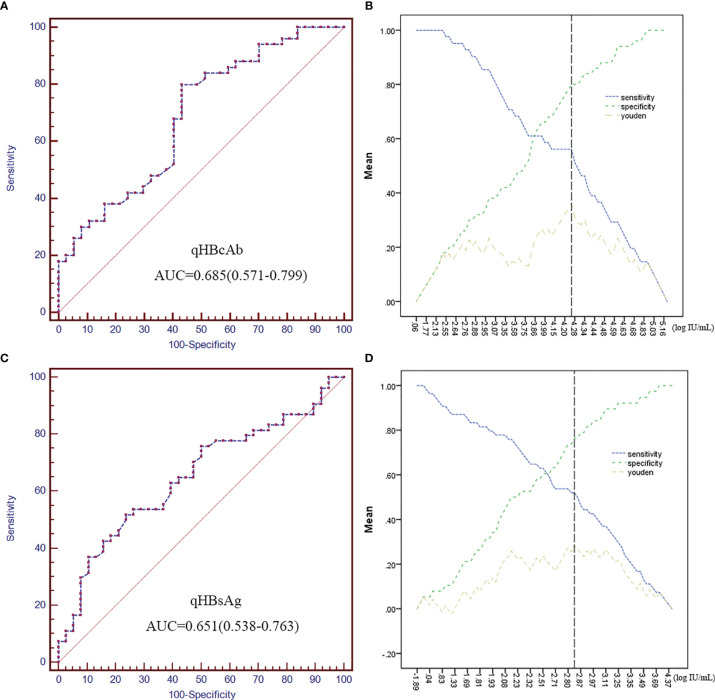
QHBcAb and qHBsAg as predictors of HBsAg recurrence. **(A, C)**. Receiver operating characteristic (ROC) analyses of qHBcAb and qHBsAg as predictors of HBsAg recurrence at baseline. **(B, D)**. Curves of the specificity, sensitivity and Youden index for qHBcAb and qHBsAg. The position of the black vertical dotted line shows the maximum Youden index that was used to predict HBsAg recurrence.

The ability of qHBcAb and qHBsAg at baseline to predict HBsAg recurrence was analyzed using ROC curves to compare HBsAg recurrence and sustained HBsAg loss groups (**Figures 2A, C**). The area under the curves (AUCs) of qHBcAb and qHBsAg were 0.685 (95% confidence interval, 0.577 - 0.799) and 0.651 (95% confidence interval, 0.538 - 0.763), respectively. Using the Youden index (**Figures 2B, D**), we predicted that when qHBcAb was higher than 4.25 log_10_ IU/mL and qHBsAg was lower than 2.82 log_10_ IU/mL, sustained HBsAg loss would occur more readily after LT.

### Combined qHBcAb With qHBsAg as Predictors of HBsAg Recurrence

**Figure 3:**
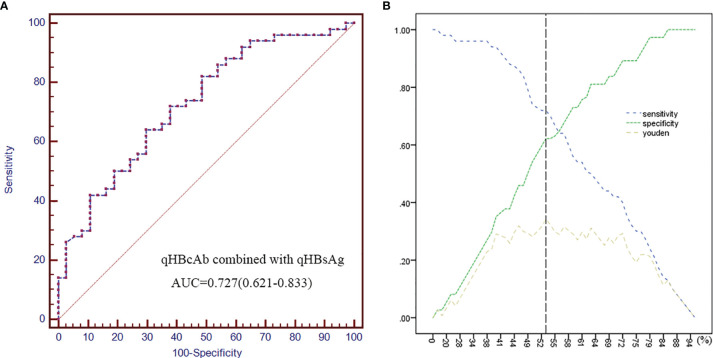
Combined qHBcAb with qHBsAg as predictors of HBsAg recurrence. **(A)** The ROC curve of combined qHBcAb and qHBsAg at baseline. **(B)** Curves of the specificity, sensitivity and Youden index. The position of the black vertical dotted line shows the maximum Youden index that was used to predict HBsAg recurrence.

As shown in **Figure 3**, the combination of qHBsAg and qHBcAb was a stronger predictor of HBsAg recurrence than individual biomarkers at baseline. The cutoff values of 1.81 log10 IU/mL and 3.68 log10 IU/mL at baseline for qHBsAg and qHBcAb, respectively, had sensitivity of 72.0%, specificity of 62.2%, PPV of 72.0% and NPV 62.2%, with an AUC value of 0.727 (0.621, 0.833; 95% CI) (**Supplementary Table 1**).

### Comparison of qHBcAb and qHBsAg at Baseline Using Kaplan-Meier Method

**Figure 4:**
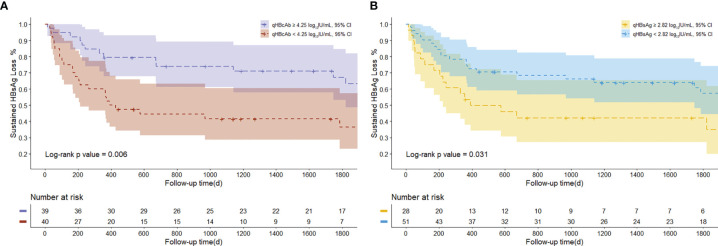
The Kaplan-Meier curve of HBsAg conversion rates at the last time of follow-up. **(A)** The proportion of patients with sustained HBsAg loss according to qHBcAb at baseline. **(B)** The proportion of patients with sustained HBsAg loss according to qHBsAg at baseline.

**Figures 4A, B** show the comparison of HBsAg recurrence rates at the time of the last follow-up by Kaplan-Meier method. Patients who experienced high a risk of HBsAg recurrence had lower qHBcAb (qHBcAb < 4.25 log_10_ IU/mL) and higher qHBsAg (qHBsAg > 2.82 log_10_ IU/mL) levels than those who achieved sustained HBsAg loss (*p* = 0.006 and 0.031, respectively) (**Figure 4**).

## Discussion

HBV infection is associated with progression to hepatocellular carcinoma or other end-stage liver diseases, and LT is the only curative therapeutic method (21). However, increased liver transplantation failure rate and decreased patient survival rate in these patients are correlated with HBV reinfection and recurrence of hepatocellular carcinoma (22). HBIG and antiviral treatments are administered alone or in combination to prevent reinfection with HBV after LT (23). HBIG and nucleos(t)ide analogs can prevent reinfection with HBV through different mechanisms. HBIG eliminates circulating virus particles and induces antiviral and immune-mediated functions (24), while nucleos(t)ide analogs directly inhibit the process of viral reverse transcription, thus reducing viral load (25). The treatment of such patients aims to increase the “sustained HBsAg loss” rate during the period of follow-up. The HBV reinfection rate in patients post-LT was approximately 10% after they received combined prophylactic treatment with HBIG and NAs (26).

A newly developed assay based on double-sandwich immunoassay technology to measure qHBcAb showed higher sensitivity and specificity than those based on competitive/inhibitory principles (19, 27). Several studies have focused on the use of qHBcAb to evaluate the antiviral therapeutic effect in patients with CHB disease. Yuan et al. revealed that qHBcAb was closely correlated with hepatic inflammatory activities and can serve as a new marker of antiviral treatment response in CHB patients (17, 28). However, the measurement of qHBcAb to determine the risk of HBsAg recurrence after LT has not been reported. This study aimed to analyze qHBcAb and other serological viral markers to identify the predictors of HBsAg recurrence after LT.

Here, ROC curve was used to predict HBsAg recurrence, we found that qHBcAb was a stronger predictor than qHBsAg of HBsAg recurrence after LT due to its larger AUC value. In addition, Cox regression analysis was performed to identify the potential risk factors for HBsAg recurrence after LT; qHBcAb and qHBsAg levels at baseline had more satisfactory HR scores than other markers, confirming the predictive ability of qHBcAb and qHBsAg for HBsAg recurrence after LT.

Furthermore, on the basis of cutoff values obtained from the ROC analysis at baseline, we used the different ranges of qHBcAb and qHBsAg levels to evaluate the sustained HBsAg loss rate by Kaplan-Meier analysis. We found that low qHBcAb and high qHBsAg levels were associated with HBV reinfection.

Circulating HBsAg correlates with the presence of covalently closed circular DNA (cccDNA) (29), and is a key marker of infection, a high HBsAg level indicates a high hepatitis B viral load *in vivo*. Therefore, patients with high serum qHBsAg titers are associated with high a probability of HBsAg recurrence after LT. However, the mechanism underlying the predictive value of HBcAb titer remains unclear. Chan et al. revealed that hepatitis B core antigen (HBcAg), a viral nucleocapsid protein, is the most immunogenic component of HBV (30). Zgair et al. demonstrated that HBcAb plays an important role in inhibiting HBV through the hepatocytotoxic effect of anti-HBc-secreting B cells (16). Thus, a high qHBcAb level can represent a strong immune response to HBV and may be the reason for sustained HBsAg loss in patients with a high titer of qHBcAb at baseline.

In this study, some novel results were obtained in LT patients from baseline to the last follow-up period, and a higher qHBcAb level before LT corresponded with sustained HBsAg loss. These conclusions may be meaningful for adjusting the duration of antiviral medicine usage after LT in patients with different qHBcAb and qHBsAg levels. However, this study has some limitations. First, the results were based on a small sample size, possibly limiting the accuracy of results. Second, related data on HBV genotype and mutation were not acquired; this may play potential roles during treatment (31–33). Third, the markers mentioned in this study were only serum markers, while other immunological markers and liver tissues were not analyzed. In summary, this is the first study to describe qHBcAb as a marker to predict the treatment response of LT patients. The measurement of qHBcAb could potentially assist surgeons in identifying patients who might benefit from specific antiviral treatments after LT.

## Data Availability Statement

The original contributions presented in the study are included in the article/**Supplementary Material**. Further inquiries can be directed to the corresponding author.

## Ethics Statement

The studies involving human participants were reviewed and approved by Ethics Committees of the First Affiliated Hospital, College of Medicine, Zhejiang University and 1964 Helsinki declaration and its later amendments. The patients/participants provided their written informed consent to participate in this study.

## Author Contributions

YC and QY planned and designed the study. BL and GM performed the experiments and wrote the paper. FL and FX performed serum qHBsAg, qHBeAg, HBcAb, and HBV DNA measurement experiments. YD, YT, and JZ performed the serum qHBcAb measurement. All authors contributed to the article and approved the submitted version.

## Funding

This work was supported by the National 863 Program for the Biological and medical technology (no. 2011AA02A100) and the National Key Programs for Infectious Diseases of China (no. 2012ZX10002005).

## Conflict of Interest

The authors declare that the research was conducted in the absence of any commercial or financial relationships that could be construed as a potential conflict of interest.

## Publisher’s Note

All claims expressed in this article are solely those of the authors and do not necessarily represent those of their affiliated organizations, or those of the publisher, the editors and the reviewers. Any product that may be evaluated in this article, or claim that may be made by its manufacturer, is not guaranteed or endorsed by the publisher.
