# Supplementary material for: Baseline Quantitative Hepatitis B Core Antibody Titer Is a Predictor for Hepatitis B Virus Infection Recurrence After Orthotopic Liver Transplantation
Source: Front Immunol. 2021 Oct 27;12:710528. doi: 10.3389/fimmu.2021.710528 (PMC8579009; doi:10.3389/fimmu.2021.710528)
Supplement: Supplementary file 1 [file DataSheet_1.docx]

sFig. 1


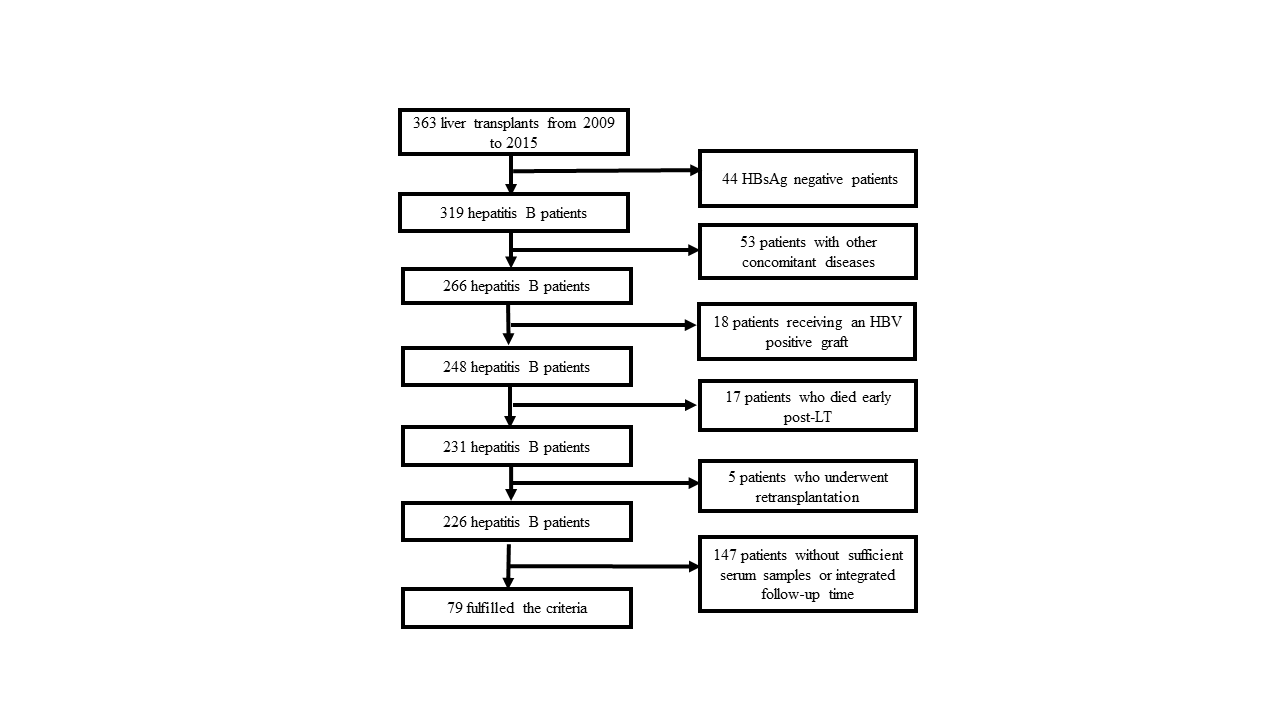


sFig. 1 Flowchart of patient selection.

sFig.2


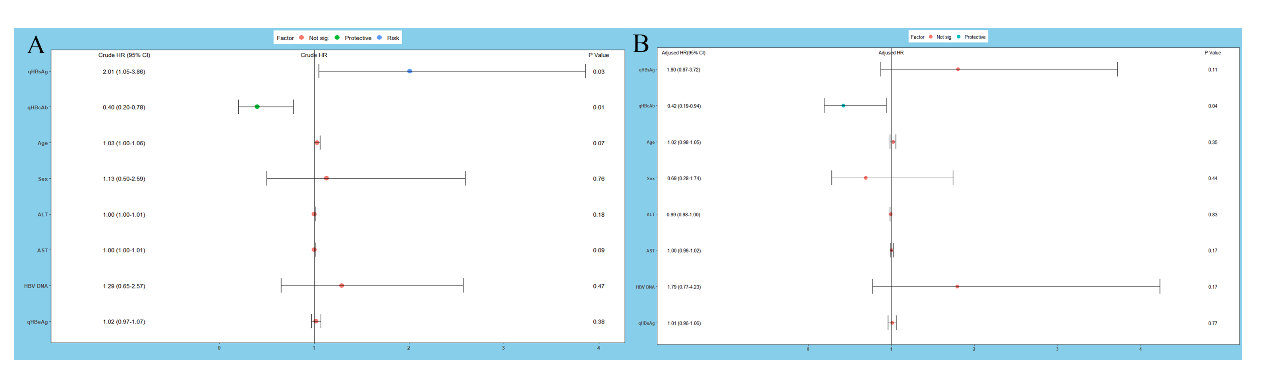


sFig.2 Risk factors of HBV recurrence after LT

sTable 1

| Supplementary table 1 Performance of baseline qHBsAg and qHBcAb level in predicting HBsAg clearance in LT cohorts | | | | | | | | |
| --- | --- | --- | --- | --- | --- | --- | --- | --- |
|  |  |  |  |  |  |  |  |  |
| Cohorts | cutoff value | AUROC | Sensitivity | Specificity | +LR | -LR | +PV | -PV |
| qHBsAg | ≤2.82 log_10_ IU/mL | 0.687 | 51.85 | 76.32 | 2.19 | 0.63 | 75.70 | 52.70 |
| qHBcAb | ≥4.25 log_10_ IU/mL | 0.651 | 80.00 | 56.10 | 1.82 | 0.36 | 69.00 | 69.70 |
| Combined qHBsAg | qHBcAb≥3.68 log_10_ IU/mL | 0.727 | 72.00 | 62.16 | 1.90 | 0.45 | 72.00 | 62.20 |
| with qHBcAb | qHBsAg≤1.81log_10_ IU/mL |  |  |  |  |  |  |  |

AUROC, area under the receiver operator characteristic curve; −LR, negative likelihood ratio; +LR, positive likelihood ratio; −PV, negative

predictive value; +PV, positive predictive value

sTable 2

Biomarker characteristics of donor at baseline

| Biomarker Characteristics of Donor | HBsAg recurrence | Sustained HBsAg loss | *p* |
| --- | --- | --- | --- |
|  | in follow-up time | after LT |  |
|  | (n=37) | (n=42) |  |
| HBcAb (positive%) | 14 (37.8%) | 16 (38.1%) | *ns* |
|  |  |  |  |
| HBsAb (positive%) | 15 (40.5%) | 22 (52.4%) | *ns* |
|  |  |  |  |
| HBsAg (positive%) | 0 (0%) | 0 (0%) | *ns* |
|  |  |  |  |

ns: no significant
